# The Antimicrobial and Antiviral Activity of Polyphenols from Almond (*Prunus dulcis* L.) Skin

**DOI:** 10.3390/nu11102355

**Published:** 2019-10-03

**Authors:** Maria Musarra-Pizzo, Giovanna Ginestra, Antonella Smeriglio, Rosamaria Pennisi, Maria Teresa Sciortino, Giuseppina Mandalari

**Affiliations:** 1 Department of Chemical, Biological, Pharmaceutical and Environmental Science, University of Messina, Viale SS. Annunziata, 98168 Messina, Italy; mmusarrapizzo@unime.it (M.M.-P.); giovanna.ginestra@unime.it (G.G.); asmeriglio@unime.it (A.S.); rosypennisi@siitm.org.cn (R.P.); gmandalari@unime.it (G.M.); 2 Shenzhen International Institute for Biomedical Research, 140 Jinye Ave. Building A10, Dapeng New District, Shenzhen 518116, Guangdong, China

**Keywords:** *S. aureus*, antimicrobial, herpes simplex virus, antiviral, almonds, polyphenols

## Abstract

Due to their antimicrobial and antiviral activity potential in vitro, polyphenols are gaining a lot of attention from the pharmaceutical and healthcare industries. A novel antiviral and antimicrobial approach could be based on the use of polyphenols obtained from natural sources. Here, we tested the antibacterial and antiviral effect of a mix of polyphenols present in natural almond skin (NS MIX). The antimicrobial potential was evaluated against the standard American Type Culture Collection (ATCC) and clinical strains of *Staphylococcus aureus*, including methicillin-resistant (MRSA) strains, by minimum inhibitory concentration (MIC) and minimum bactericidal concentration (MBC). Herpes simplex virus type I was used for the antiviral assessment of NS MIX by plaque assay. Furthermore, we evaluated the expression of viral cascade antigens. NS MIX exhibited antimicrobial (MIC values of 0.31–1.25 mg/ml) and antiviral activity (decrease in the viral titer ** *p* < 0.01, and viral DNA accumulation * *p* < 0.05) against *Staphylococcus aureus* and HSV-1, respectively. Amongst the isolated compounds, the aglycones epicatechin and catechin showed the greatest activity against *S. aureus* ATCC 6538P (MIC values of 0.078–0.15 and 0.15 mg/ml, respectively), but were not active against all the other strains. These results could be used to develop novel products for topical use.

## 1. Introduction

A number of studies have indicated that phytochemicals present in almond (*Prunus dulcis* L.) skins are associated with beneficial effects on chronic conditions and certain types of cancer [1,2]. The distribution of flavonoids and phenolics in almond skins has previously been investigated: The most represented flavonoids are (+)-catechin, (-)-epicatechin, kaempferol, and isorhamnetin, the latter as 3-*O*-rutinoside or 3-*O*-glucoside [3,4]. Almond skin polyphenols were effective against scavenging free radicals and inducing quinone reductase in a dose-dependent way [5]. We have previously demonstrated the antimicrobial potential of polyphenol-rich extracts from natural almond skins (NS) and their main isolated compounds: natural and blanched (resulted from industrial blanching) almond skin extracts were active against *Listeria monocytogenes* and *Staphylococcus aureus*, whereas *Salmonella enterica* and *Helicobacter pylori* were sensitive to natural almond skins [6,7,8]. The acquisition of antibiotic resistance of the *S. aureus* and methicillin-resistant (MRSA), which are invasive Gram-positive human pathogens responsible for a range of infections, as well bacteremia and toxic shock syndrome [9], currently represents another virulence factor of these strains. In addition, the antiviral effect of natural almond skins was also tested against herpes simplex virus type 2 (HSV-2) and herpes simplex virus type 1 (HSV-1) [10,11]. The most accepted therapies to treat HSV-1 infection are based on the use of inhibitors of viral DNA replication. In particular, acyclovir is routinely used as a treatment of this viral infection. Nevertheless, due to an increase in viral mutations, as well as the incapability of the acyclovir to protect against the virus in the latent state, reoccurrence of the disease results in humans. In addition, HSV-1 infections are sometimes fatal for children and immunocompromised people. The above data and the occurrence of drug-resistant viral strains leads to studies aimed to develop novel anti-herpetic compounds. Indeed, in recent years, numerous effective anti-HSV-1 compounds are derived from plants. Several researchers have shown that natural compounds such as phenols and flavonoids, extracted from different plants, have antiherpetic properties. In particular, polyphenol-rich almond skin extracts have displayed many pharmacological properties, including anti-inflammatory and anti-herpetic activity [11,12,13]. 

Taking into account these evidences, the aim of the present work was to assess the antimicrobial and the anti-herpetic effect of a mix of pure polyphenol compounds present in almond skins (NS MIX). Thus, both individual compounds and NS MIX were tested against ATCC and clinical strains of *S. aureus*, including methicillin-resistant (MRSA) strains, and HSV type I. The antimicrobial and antiviral effect of a polyphenolic mix obtained from almond skins could help develop new formulations for topical use.

## 2. Materials and Methods

### 2.1. Preparation of Polyphenols Mix

NS MIX was prepared according to the natural skin (NS) polyphenols extract composition [11]. The most abundant compounds (HPLC-grade, purity ≥ 99%) identified and quantified in the previous work (catechin, naringenin-7-*O*-glucoside, kaempferol-3-*O*-glucoside, epicatechin, isorhamnetin-3-*O*-rutinoside, and isorhamnetin-3-*O*-glucoside) were acquired from Extrasynthese (Genay, France) and mixed in the same proportion in which they are present in the NS extract (28:20:17:15.5:10:9.5 *w/w*) [11]. The NS MIX was dissolved in DMSO (stock solution 100 mg/mL) and properly diluted in order to obtain a final DMSO concentration ≤0.01%.

### 2.2. Microbial Strains and Culture Conditions

The following strains, obtained from the University of Messina’s in-house culture collection (Messina, Italy), were used: *S. aureus* ATCC 6538P, *S. aureus* ATCC 43300 (MRSA), four clinical strains of *S. aureus* obtained from pharynges (strains 526, 530, 808, 814), two clinical strains of *S. aureus* obtained from duodenal ulcers (strains 8, 14), and three clinical strains of *S. aureus* obtained from hip prostheses (strains 6, 32, 84). All tested strains were resistant to the combination imipenem–cilastatin, whereas the MRSA ATCC strain 43300 and strain 84 were also resistant to clarithromycin. Three (strains 526, 530, and 815) out of the 4 clinical strains from pharynges were MRSA. All strains were cultured in Mueller-Hinton Broth (MHB, Oxoid, CM0405) at 37 °C (24 h). 

### 2.3. Susceptibility Assay

The minimum inhibitory concentration (MIC) and the minimum bactericidal concentration (MBC) of NS MIX, the individual polyphenols and the two antibiotics vancomycin and teicoplanin (Sigma, Milan, Italy) were determined using a broth microdilution method according to the Clinical and Laboratory Standards Institute [14]. MIC values were defined as the lowest extract concentrations with no bacterial growth after the incubation. MBCs were determined by seeding 20 µL from all clear MIC wells onto Mueller-Hinton agar (MHA, Oxoid, Milan, Italy) plates. The MBC was defined as the lowest concentration that killed 99.9% of the final inocula after 24 h incubation at 37 °C. All assays were done in triplicate.

### 2.4. Cell Cultures and Virus

Vero cell lines were cultured in Eagle’s minimum essential medium (EMEM, Lonza, Belgium), with 6% fetal bovine serum (FBS, Euroclone) and a mixture of penicillin (100 U/ml) and streptomycin (100 µg/ml) (Lonza, Belgium). The cell lines were maintained at 37 °C under 5% CO_2_. The prototype Herpes simplex virus type 1 strain F (HSV-1) was a generous gift from Professor Bernard Roizman (University of Chicago, IL, USA). Viral stocks were obtained from Vero cells infected with HSV-1.

### 2.5. Cell Viability Assay

The cell viability of samples treated with NS MIX was measured by using the ViaLightTM plus cell proliferation and cytotoxicity bioassay kit, according to the manufacturer’s instructions (Lonza Group Ltd., Basel, Switzerland). Vero cells were grown in 96-well plates and treated with NS MIX at different concentrations (1.6, 0.8, 0.6, 0.4, 0.2, and 0.1 mg/mL). DMSO was used as negative control. After 72 h the ATP levels was quantified in a GloMax Multi Microplate Luminometer (Promega Corporation, 2800 Woods Hollow Road Madison, WI, USA). The luminescence value was converted into the cell proliferation index (%), as previously reported [11]. The 50% cytotoxic concentration (CC_50_) was calculated from concentration-effect curves by using non-linear regression analysis. The GraphPad Prism 6 software was used for data analysis. The results are the means (± SD) of three independent experiments.

### 2.6. Plaque Reduction Assay 

The antiviral effect of NS MIX was evaluated through plaque reduction assay on Vero cells treated with the following concentrations: 0.4, 0.2, and 0.1 mg/mL. The virus inoculum was diluted to yield 100 plaques/100 µL. Confluent monolayers of VERO cells were cultured in 6 multiwall plates, pre-treated with NS MIX for 1h, after which the cells were infected with 100 µl of each dilution for 1 h at 37 °C under gentle shaking. Then, the viral inoculum was removed and the cells were incubated for 72 h with medium containing 0.8% methylcellulose in the presence of NS MIX at the indicated concentrations. DMSO was used as negative control. The acyclovir (Acv) was used as a positive control. The final drug concentrations of Acv were 1, 10, and 20 μM. The cells were stained with crystal violet and the plaques were visualized at 10× magnification with an inverted microscope (Leica DMIL). The data were analyzed as means of triplicate ± SD for each dilution.

### 2.7. HSV-1 Infection on Vero Cells

VERO cells were pre-treated with 0.4 mg/mL of the NS MIX for 1 h and thereafter the cells were either uninfected (mock) or infected with HSV-1 at multiplicity of infection (MOI) of 1 for 1 h at 37 °C under gentle shaking. The inoculum was then removed and the cells were treated or not with the NS MIX (0.4 mg/mL). After 24 h the cells were collected and subjected to immunoblotting analysis for the detection of the viral antigens and the quantification of viral DNA.

### 2.8. Antibodies

Anti-GAPDH, anti-ICP0, and anti-UL42 antibodies were purchased from Santa Cruz Biotechnology (Santa Cruz, CA, United States). Monoclonal antibody against Us11 was kindly provided by Professor Bernard Roizman. Anti-rabbit and anti-mouse IgG HRP (horseradish peroxidase) secondary antibodies were also from Santa Cruz Biotechnology.

### 2.9. Protein Extractions and Immunoblot Analysis

Proteins extraction was performed by using SDS sample buffer 1X (62.5 mM Tris-HCl pH 6.8; DTT 1 M; 10% glycerol; 2% SDS; 0.01% Bromophenol Blue). An equal amount of protein extract was subjected to SDS gel electrophoresis (SDS-PAGE) and transferred to nitrocellulose membranes (Bio-Rad Life Science Research, Hercules, CA). Membranes were incubated with the primary antibody overnight at 4 °C and then for 1 h at room temperature (RT) with secondary antibodies. The chemiluminescent detection was performed by using western HRP substrate (Merk, Millipore) as a chemiluminescent substrate. The housekeeping GAPDH protein was used as loading control.

### 2.10. DNA Extraction and Quantitative Real-Time RT-PCR

Samples were collected and resuspended in TRIzol^®^ (Life Technologies, CA, United States) for DNA extraction, according to the manufacturer’s instructions. The DNA extraction and absolute quantification using specific TaqMan probe was performed as described previously [15]. The amplification of viral DNA was carried out in a 25 μL reaction mixture containing: DNA (1µg), HSV-1 forward (0.5 μM), and HSV-1 reverse (0.5 μM) primers (Fw 5’-catcaccgacccggagagggac; Rev 5’-gggccaggcgcttgttggtgta), TaqMan probe (1µM) (5’-6FAM-ccgccgaactgagcagacacccgcgc-TAMRA, where 6FAM is 6-carboxyfluorescein and TAMRA is 6-carboxytetramethylrhodamine), dNTP mix (1 µM), NH4 reaction buffer 1×, MgCl_2_ (2mM), and BIOTAQ^TM^ (5U/µL) thermostable DNA polymerase (BIO-21040 Bioline). The amplification was carried out in a Cepheid SmartCycler II System (Cepheid Europe, Maurens-Scopont, France) under the following conditions—10 min at 95 °C, 30 s at 95 °C for 40 cycles, 30 s at 55 °C, and 30 s at 72 °C, with a final cycle of 5 min at 72 °C. Each amplification run contained one negative control.

### 2.11. Statistical Analysis

Student’s *t*-test was used to perform the statistical analysis of the data. Quantitative densitometry analysis of immunoblot bands intensity was performed with TINA software (version 2.10, Raytest, Straubenhardt, Germany). The data analysis and the graphical representations were produced with the GraphPad Prism 6 software (GraphPad Software, San Diego, CA, USA). Data are expressed as a mean (± SD) of at least three experiments and asterisks (*, **, and ***) indicate the significance of *p*-values less than 0.05, 0.01, and 0.001, respectively.

## 3. Results

### 3.1. Antimicrobial Activity of Polyphenols

The MIC and MBC values of NS MIX and the two antibiotics used as positive controls against all the tested strains are reported in Table 1. NS MIX was effective against all tested strains, ATCC 6538P being the most sensitive (complete inhibition achieved with a concentration of 0.31 mg/mL), followed by strain 6 (complete inhibition achieved with a concentration of 0.62 mg/mL). The effect was bacteriostatic rather than bactericidal. As expected, the ATCC MRSA strain 43300 was more resistant that the ATCC 6538P.

The MICs of the five most abundant almond skin flavonoids are reported in Table 2. The aglycones catechin and epicatechin showed the greatest activity against *S. aureus* ATCC 6538P, but were not active against all the other strains. In agreement with our previous investigation [6], isorhamnetin-3-*O*-rutinoside was the least effective against *S. aureus*. As for the NS MIX, the activity was bacteriostatic rather than bactericidal. 

No bactericidal activity was detected when individual compounds were used.

### 3.2. Cytotoxicity and Antiviral Activity Tests

#### Viability Assay

**Figure 1:**
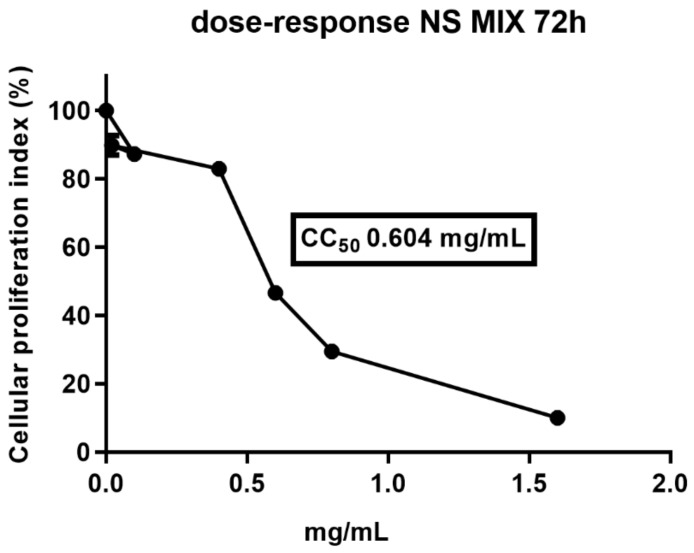
Viability of Vero cells treated with NS MIX. Vero cells were incubated with 1.6, 0.8, 0.6, 0.4, 0.2, and 0.1 mg/mL of NS MIX for 72 h. DMSO was used as a negative control. Cells were then collected and their viability was determined using the ViaLight™ plus cell proliferation and cytotoxicity bioassay kit (Lonza Group Ltd, Basel, Switzerland). The luminescence value was converted into cellular proliferation index as described in Materials and Methods. Results represent the mean of three biological independent experiments ± SD.

Our published data [11] indicated that 0.4 mg/mL of natural polyphenol-rich almond skin extract resulted in a >90% decrease of viral titer of HSV-1 with non-toxic effect on Vero cells [11]. Based on these data, a cell viability assay was carried out in order to evaluate the effect of NS MIX on Vero cells. The cells were treated with different concentrations of NS MIX (1.6, 0.8, 0.6, 0.4, 0.2, and 0.1 mg/mL) for 72h. The cell proliferation index (%) was calculated on the basis of ATP degradation, as reported in Material and Methods. As shown in Figure 1, treatment with NS MIX results in a decrease of the cell proliferation index at 72 h of treatment using the concentration ranging between 0.6 and 1.6 mg/mL only. No cytotoxicity effect was found at concentrations of 0.4, 0.2, and 0.1 mg/mL. The 50% cytotoxic concentration (CC_50_) was 0.604 mg/mL.

### 3.3. Antiviral Activity of Polyphenols Against HSV-1

We investigated whether treatment with NS MIX interfered with viral replication. The antiviral activity was evaluated by plaque reduction assay, the analysis of viral protein expression of representative *αlpha* ICP0, *ßeta* ICP8, and *γamma* Us11 viral proteins and quantification of viral DNA by qPCR.

#### 3.3.1. Plaque Assay

**Figure 2:**
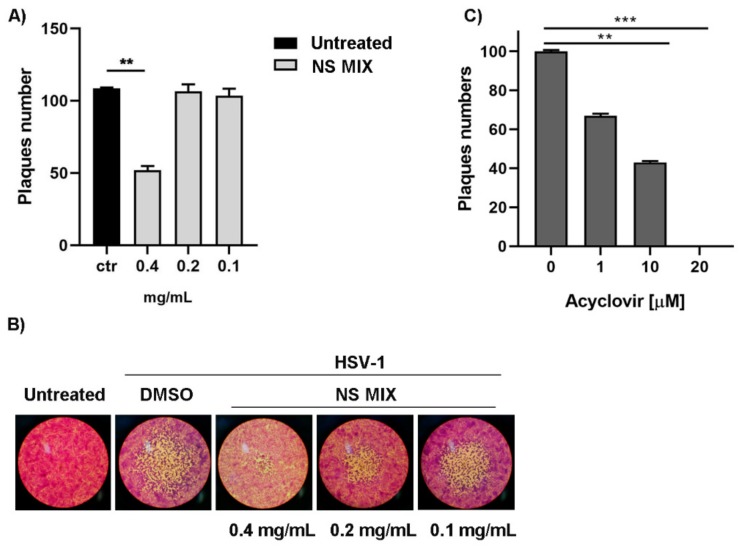
Antiviral activity of NS MIX. Vero cells were pre-treated with 0.4, 0.2, and 0.1 mg/mL of NS MIX for 1 h and the cells were then infected with Herpes simplex virus type 1 strain F (HSV-1) for 1 h at 37 °C under gentle shaking. After incubation, the inoculum was removed and the cells were covered with medium containing 0.8% methylcellulose in the presence of NS MIX at 0.4, 0.2, and 0.1 mg/mL, separately. After three days the cells were fixed and stained with crystal violet and the plaques were visualized with an inverted microscope. (**A**) Plaque reduction assay following the NS MIX treatment. (**B**) Plaque morphological change due to the NS MIX treatment. Results are the mean of three biological independent experiments ± SD for each dilution. (**C**) Antiviral activity of acyclovir was tested in Vero cells infected with HSV-1 and incubated with medium containing 0.8% methylcellulose in the presence of acyclovir at 1, 10, and 20 μM. The cells were stained with crystal violet for plaque counting and detection. Data are expressed as a mean (± SD) of at least three experiments and asterisks (**, ***) indicate the significance of *p*-values less than 0.01, and 0.001, respectively.

A significant decrease in the viral titer and plaques morphology was found after incubation with NS MIX at the concentration of 0.4 mg/mL (** *p* < 0.01). No reduction was observed at the concentrations of 0.2 and 0.1 mg/mL (Figure 2A,B). The highest concentration of NS MIX (1.6, 0.8, and 0.6 mg/mL) was not unrolled, since associated to a cytotoxic effect on the cells and a consequent inhibition of viral replication. No antiviral activity was detected when individual compounds were used (data not shown). Acyclovir was used as positive control, data demonstrated the total inhibition of viral replication at concentration of 20 µM. To note, the effectiveness of the NS MIX of 0.4 mg/mL was comparable to those obtained with acyclovir treatment at a concentration of 10 µM.

#### 3.3.2. Expression of Viral Antigens and Quantification of Viral DNA

**Figure 3:**
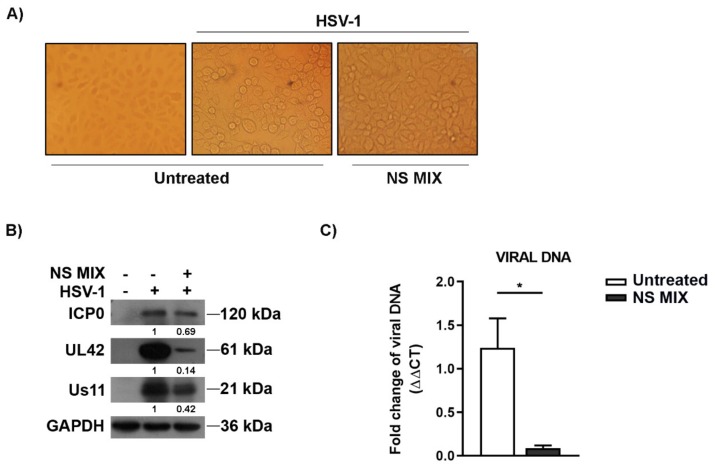
NS MIX affected the expression of viral antigens and HSV-1 replication. (**A**) Normal phase contrast inverted micrographs of Vero cells treated with 0.4 mg/mL of NS MIX. The cells were pre-treated with NS MIX for 1 h at 37 °C, then cells either mock infected or infected with HSV-1 at multiplicity of infection (MOI) of 1 for 1 h and incubated in presence of 0.4 mg/mL of the NS MIX for 24 h. (**B**) Immunoblot analysis was performed to detect α (ICP0), β (UL42), and γ (US11) viral proteins. GAPDH protein was used as loading control. Band density indicated in the figure was determined with the TINA program (version 2.10, Raytest, Straubenhardt, Germany) and expressed as the fold change over the housekeeping gene GAPDH. (**C**) Relative quantization of viral DNA was performed using real-time quantitative PCR and analyzed by the comparative Ct method (ΔΔCt). Data are expressed as a mean (± SD) of at least three experiments and asterisk (*) indicate the significance of *p*-values less than 0.05.

Next, we investigated whether the production of viral DNA and viral proteins cascade expression were also affected by the treatment with NS MIX. As shown in Figure 3A, treatment with NS MIX clearly reduced the cytopathic effect following infection with HSV-1. In addition, treatment with NS MIX (0.4 mg/mL) reduced the expression of the viral proteins ICP0, UL42, Us11 (Figure 3B), and viral DNA accumulation (* *p* < 0.05) compared to the untreated infected cells (Figure 3C).

## 4. Discussion

There is a clear need for the discovery of novel natural compounds against viruses or pathogenic bacteria. The use of a variety of natural extracts derived from plants, as medicine use, has been tested for the treatment of various diseases. Indeed, researchers from different fields are investigating plants with the aim of discovering molecules able to treat bacterial and viral infections. Generally, pathogenic viruses as well as bacteria are more and more difficult to treat with existing drugs. In particular, antibiotic or antiviral resistance is increasing worldwide in both outpatients as well as hospitalized patients. We have previously demonstrated the antimicrobial and antiviral potential of polyphenol-rich extracts from almond skins [7,11]. In the present study, we have demonstrated for the first time that the main compounds identified in almond skin extracts have both antimicrobial and antiviral activity. This effect could be used to develop novel formulations for topical application.

The *S. aureus* strains used here have been recently characterized in terms of lipid profile and their correlation with antibiotic resistance and hydrophobicity [16]. A distinction in two clusters based on the amount and type of bacterial lipids identified was obtained, which correlated to the antibiotic resistance, the strains origin, and the cell-surface hydrophobicity. Strain 6 isolated from hip prostheses was the most sensitive to NS MIX, followed by *S. aureus* strains obtained from pharynges (strain 526, 808, 814). Amongst the isolated compounds, isorhamnetin-3-*O*-rutinoside and kaempferol-3-*O*-glucoside were the most active against the clinical strains, followed by naringenin-7-*O*-glucoside. All strains from hip prostheses (6, 32, 84) were more sensitive to isorhamnetin-3-*O*-rutinoside, kaempferol-3-*O*-glucoside, and naringenin-7-*O*-glucoside, indicating a possible correlation between site of infection and mechanism of action. Generally, the effect of phenolic compounds on bacterial cells involves damage of the cellular membrane, binding of the cell wall, enzyme inactivation, DNA damage [17]. The antimicrobial mechanism of action of naringenin against *S. aureus* ATCC 6538 has recently been investigated: Its antibacterial effects might be connected with disruption of the cytoplasmic membrane and DNA targeting effects [18]. Kaempferol and myricetin were found to be potent inhibitors targeting *S. aureus* PriA helicase [19]. Another study reported the effect of kaempferol on the primary attachment phase of biofilm formation in *S. aureus* [20]. In combination assays, rifampicin with kaempferol or quercetin exhibited good beta-lactamase inhibitory effects (57.8% and 75.8%, respectively) against a representative isolate of *S. aureus* [21]. In a recent investigation, isorhamnetin attenuated *S. aureus*-induced lung cell injury by inhibiting alpha-hemolysin expression and; therefore, could represent a leading compound for the development of anti-virulence drugs against *S. aureus* infections [22].

Regarding the antiviral activity, different molecules, such as flavonoids from a range of plant species, have been described to display inhibitory activities on HSV replication [23,24,25]. Strong inhibition of HSV-1 and -2 was achieved by the aqueous extract of *Pelargonium sidoides*, which mainly contains simple phenolics and catechins [26]. In particular, extracts rich in polyphenols have been found to interfere with the viral particles directly through inhibition of virus attachment [11]. The anti-adhesive effect of the almond skin extract was mainly due to its capacity to alter the binding of the virus to the target cell. In this study, we have formulated a NS MIX of the most abundant compounds mixed in the same proportion present into the NS extract in order to verify its antiviral activity. However, a drastic decrease in viral infectivity was detected for HSV-1 when NS MIX was used to pre-treat the cellular substrate, but not when NS MIX formulation was used directly on the viral suspension by avoiding the viral binding. On the other hand, pre-treatment of the monolayers with NS MIX suppressed the accumulation of immediate early and early viral proteins, such as ICP0 or UL 42, as well as the viral DNA (Figure 3). Based on these data and on the literature data it is possible to hypothesize that NS MIX could interfere in HSV gene expression at early or late times of viral replication [27,28]. However, in our study it is not clear if the mechanisms of action were directed to viral structures or on the cellular environment, which does not favor an efficient viral replication. To the best of our knowledge, our study describes for the first time that NS MIX exert both anti-HSV type-1 and antimicrobial activity and could potentially be used in a topical formulation. Further studies are needed to establish possible synergistic effects with antibiotics or antivirals, aiming to develop novel agents for the treatment of both *S. aureus* and HSV-1 human infections. Lastly, the understanding of the mechanisms could help the formulation of drugs for topical use, especially for those patients who experience frequent recurrences.

## 5. Conclusions

In conclusion, our study describes for the first time that NS MIX exert both anti-HSV type-1 and antimicrobial activity and could potentially be used in a topical formulation. Further studies are needed to establish possible synergistic effects with antibiotics or antivirals, aiming to develop novel agents for the treatment of both S. aureus and HSV-1 human infections. Lastly, the understanding of the mechanisms could help the formulation of drugs for topical use, especially for those patients who experience frequent recurrences.

## Figures and Tables

**Table 1:** MICs and MBCs of MIX (expressed as mg/ml) against *S. aureus* ATCC and clinical strains.

	NS MIX		Vancomycin		Teicoplanin	
*S. aureus* Strain	MIC	MBC	MIC	MBC	MIC	MBC
ATCC 6538P	0.31	>1.25	0.31	0.62	0.15	0.15
43300	1.25	>1.25	0.31	0.62	0.31	0.31
526	1.25	>1.25	0.31	0.61	0.31	0.62
530	>1.25	>1.25	0.31–0.62	0.62	0.31–0.62	0.62
808	1.25	>1.25	0.31	0.31	0.15	0.15
814	1.25	>1.25	0.62	0.62	0.62	1.25
8	>1,25	>1.25	0.62–1.25	1.25	0.31	0.31
14	>1.25	>1.25	0.31	0.31	0.15–0.31	0.31
6	0.62	>1.25	0.31	0.62	1.25	1.25
84	>1.25	>1.25	0.62	0.62	0.62	1.25
32	>1.25	>1.25	0.62	0.62	0.31	0.31

MICs, minimal inhibitory concentrations; MBCs, minimal bactericidal concentrations. NS MIX, mix of polyphenols in natural almond skin; ATCC, American Type Culture Collection.

**Table 2:** Minimal inhibitory concentrations of almond skin flavonoids (expressed as mg/mL) against *S. aureus* ATCC and clinical strains.

*S. aureus* Strain	N-7-*O*-g	K-3-*O*-g	I-3-*O*-r	I-3-*O*-g	Cat	Epic
ATCC 6538P	1.25–0.62	0.62	0.31–0.15	1.25	0.15	0.15–0.078
43300	>1.25	>1.25	1.25	>1.25	>1.25	>1.25
526	>1.25	0.62	0.62	>1.25	>1.25	>1.25
530	>1.25	>1.25	0.62	>1.25	>1.25	>1.25
808	>1.25	>1.25	1.25-0.62	>1.25	>1.25	>1.25
814	>1.25	>1.25	>1.25	>1.25	>1.25	>1.25
8	>1.25	>1.25	>1.25	>1.25	>1.25	>1.25
14	>1.25	0.62	1.25–0.62	>1.25	>1.25	>1.25
6	1.25	0.62	0.62–0,31	>1.25	>1.25	>1.25
84	1.25–0.62	0.62	1,25–0.62	>1.25	>1.25	>1.25
32	1.25	0.62	0.31–0.15	>1.25	>1.25	>1.25

N-7-*O*-g, naringenin-7-*O*-glucoside; K-3-*O*-g, kaempferol-3-*O*-glucoside; I-3-*O*-r, kaempferol-3-*O*-rutinoside; I-3-*O*-g, isorhamnetin-3-*O*-glucoside; Cat, catechin; Epic, epicatechin. ATCC, American Type Culture Collection.
